# Signet cell rectal carcinoma with prostatic involvement detected by FAPI-04 PET-MRI fusion

**DOI:** 10.1186/s41824-023-00183-4

**Published:** 2023-12-01

**Authors:** K. Arun Prashanth, Deepti Jain, R. Manikandan, Suman Kalyan, Vimalraj Velayutham, R. Surendran

**Affiliations:** 1 Department of Nuclear Medicine, MIOT International Hospital, Chennai, India; 2 Department of Lab Services, MIOT International Hospital, Chennai, India; 3 Department of Urology, MIOT International Hospital, Chennai, India; 4 Department of Medical Oncology, MIOT International Hospital, Chennai, India; 5 Department of Hepato-Pancreato-Biliary Centre for Surgery and Transplantation, MIOT International Hospital, Chennai, India

**Keywords:** Signet cell adenocarcinoma, FAPI, Rectal adenocarcinoma, Prostate secondaries, FAPI MRI fusion, Fibroblast activation protein inhibitor PET-CT

## Abstract

A 60-year-old male patient diagnosed with mucinous adenocarcinoma of lower third of rectum underwent abdominoperineal resection and permanent colostomy followed by adjuvant chemotherapy. Response evaluation with F-18 FDG PET-CT showed a complete metabolic response. After 6 months, CEA levels started increasing and clinically a recurrence was suspected. A restaging FDG PET-CT showed no obvious malignant disease. Patient presented again within a month with complaints of urinary retention and haematuria. CEA levels were further elevated, and Ga-68 FAPI-04 (FAPI) PET-CT was performed. FAPI PET-CT revealed prostatic and seminal vesicle disease involvement. Additionally, an MRI of pelvis was done and fused with FAPI PET for confirmation of prostatic involvement.

## Key message

FAPI PET-CT can help diagnose clinically challenging difficult cases.

## Background

Primary signet ring cell carcinoma of rectum was a rare malignancy contributing about less than 1% of colon cancers with aggressive behaviour (Morales-Cruz et al. 2019). Presentation usually occurs at an advanced stage, and the tumour has a distinctive molecular pattern and poor prognosis (An et al. 2021). Signet cell adenocarcinoma was diagnosed based on the presence of 50% extracellular mucin by volume in tumour.

Prostatic involvement as metastasis by colon malignancies has been sparse in the literature (Schips et al. 2002; Youssef et al. 2011; Gupta et al. 2004; Berman et al. 1993) limited to case reports. FDG PET-CT and MR imaging has been utilised for the diagnosis of prostatic involvement by colorectal malignancies (Kang et al. 2013).

## Case presentation

A 61-year-old male presented with fistula and diagnosed to have mucinous adenocarcinoma of lower third of rectum 2 years back. Examination under anaesthesia and fistulectomy was performed. FDG PET-CT showed metabolic activity in the fistulous tract and lower rectum with no nodal or distant metastasis. He was started on capecitabine and external beam radiotherapy by IGRT technique.

After chemoradiotherapy, he underwent abdominoperineal resection (APR) with permanent colostomy surgery. Adjuvant chemotherapy with oxaliplatin and irinotecan regimen was administered after surgery. A response evaluation FDG PET-CT demonstrated a complete metabolic response. On follow-up, he was asymptomatic but CEA was 17.0 ng/ml. FDG PET-CT images did not reveal any obvious malignant disease and no definite recurrence. He presented again within a month with complains of urinary retention and haematuria. The CEA value was also increased to 68.7 ng/ml. Since no malignant disease was detected in the recent FDG, a Ga-68 FAPI-04 (FAPI) PET-CT was performed instead of FDG PET-CT. FAPI PET-CT demonstrated intense tracer uptake in prostate and seminal vesicles. Since prostatic involvement is extremely rare, additionally an MRI of prostate was done for confirmation. The FAPI PET-MRI fusion images revealed the prostatic and seminal vesicle involvement. A transurethral resection of prostate (TURP) confirmed the presence of signet cell carcinoma in the prostatic parenchyma.

Haematuria was not resolved, and he developed hydroureteronephrosis due to base of bladder involvement. A bilateral PCN procedure to relieve obstruction and chemotherapy (bevacizumab and gemcitabine) for palliation was initiated. However, he further deteriorated a few months later with sepsis, bilateral metastatic pleural effusion, and ascites due to peritoneal deposits and succumbed.

## Discussion

Signet cell adenocarcinoma, an aggressive variant, has poor prognosis than other subtypes of colorectal cancers (An et al. 2021). A case series of colorectal primary locally invading prostate had only one signet cell adenocarcinoma patient (Osunkoya et al. 2007). Our patient also presented with unusual symptoms and FAPI PET-CT helped us to diagnose prostatic involvement of signet cell adenocarcinoma of rectum. In a meta-analysis, FAPI PET-CT has shown substantially greater sensitivity for the detection of peritoneal metastatic disease (Gege et al. 2023).

Signet ring cell colorectal carcinoma comprised less than 1% of colorectal and 1.4% of rectal cancers (Hyngstrom et al. 2012). The aggressive nature of signet cell morphology resulted from its predilection for intramural spread and subsequent delay in diagnosis. Primary rectal signet cell carcinoma has an average age of 48.1 years (Chen et al. 2004) in males. According to a large database analysis, signet cell ring cell adenocarcinoma of colon rectal regions was associated with 57% higher risk of death compared to other morphologies. They also have high-grade lesions compared to non-signet variants (77.2% vs 20%).

Signet colorectal carcinoma characteristic had peritoneal seeding and less hepatic metastasis. Specifically, rectal signet carcinoma has 83% peritoneal metastasis. The postulated reason was reduced expression of E-cadherin which leads to loss of epithelial differentiation and invasion. Peritoneal involvement leads to rapid progression, poor prognosis despite systemic and intraperitoneal chemotherapy (Khan et al. 2017).

Metastatic disease to the prostate was by contiguous spread and rarely by haematological dissemination. There were very few case reports for involvement of prostate by colorectal malignancies (Khan et al. 2017; Seog et al. 2022; Park et al. 2020). Few patients also presented with rising PSA triggering suspicion of secondary prostatic malignancy and masquerading the locoregional prostatic involvement.

**Fig. 1:**
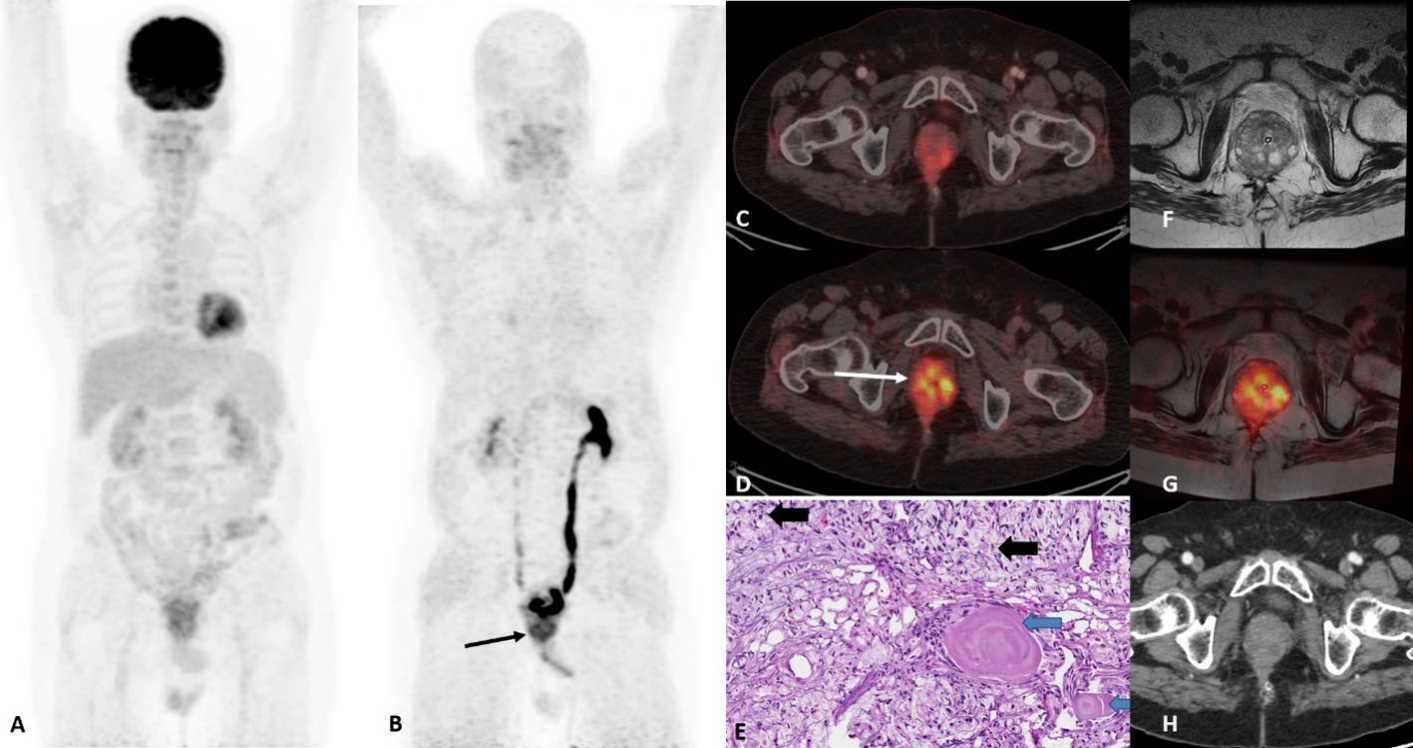
**A** FDG MIP, **B** MIP image of FAPI shows focus of uptake below the bladder (slender black arrow). **C** and **D** are axial fused images of FDG and FAPI PET-CT, respectively. The fused axial images of FAPI clearly show increased uptake (SUVmax-6.8) in prostate compared to FDG which shows only mild uptake. **E** H & E at 200× magnification image revealed normal prostate gland with corpora amylacea highlighted by blue arrows and tumour cells with signet ring morphology highlighted by bold black arrows, thus confirming the prostatic involvement by signet cell adenocarcinoma. **F** axial MRI and **G** FAPI MRI fusion images which show involvement of prostate. Seminal vesicles and base of bladder were also involved; (not shown **H**) contrast-enhanced diagnostic CT image which shows no significant abnormality

FAPI, a fibroblast activation protein inhibitor, was shown to be superior compared to conventional modalities for colorectal malignancies (Seog et al. 2022). FAPI was able to detect tiny peritoneal metastases which can be missed by FDG PET-CT. In our experience, use of FAPI PET in domains where FDG is known to have lesser sensitivity yielded better results like in gastrointestinal malignancies (Prashanth et al. 2023). In this case, FDG was negative despite rise in tumour markers. FAPI PET-CT resulted in the diagnosis of prostatic involvement which was the cause of haematuria. TURP biopsy and MRI fusion confirmed the findings of FAPI PET-CT, and patient was initiated on chemotherapy. In conclusion, FAPI PET-CT and PET-MRI fusion helped to diagnose an extremely rare involvement of prostate by signet cell rectal adenocarcinoma (Fig. 1).

## Data Availability

The datasets used and/or analysed during the current study are available from the corresponding author on reasonable request.

## Abbreviations

FAPI: Ga-68—fibroblast activation protein inhibitor-04
PET: Positron emission tomography
CT: Computed tomography
SUV: Standardised uptake value
FDG: 18-F fluorodeoxyglucose
MRI: Magnetic resonance imaging
H & E: Haematoxylin and eosin
CEA: Carcinoembryonic antigen
HPE: Histopathological examination
PCN: Percutaneous nephrostomy
IGRT: Image-guided radiotherapy
